# Multifocality as a marker of aggressiveness in medullary thyroid carcinoma: a retrospective cohort analysis of lymph node metastasis and recurrence

**DOI:** 10.1093/oncolo/oyag065

**Published:** 2026-02-28

**Authors:** Yidan Lu, Ziyi Chen, Jing Yang, Tian Jiang, Na Feng, Jincao Yao, Di Ou, Zhiyan Jin, Liyu Chen, Chen Yang, Dong Xu, Lingyan Zhou

**Affiliations:** Department of Diagnostic Ultrasound Imaging & Interventional Therapy, Zhejiang Cancer Hospital, Hangzhou Institute of Medicine (HIM), Chinese Academy of Sciences, Hangzhou, Zhejiang 310022, China; Department of Ultrasound, Taizhou Cancer Hospital, Taizhou, Zhejiang 317502, China; Department of Diagnostic Ultrasound Imaging & Interventional Therapy, Zhejiang Cancer Hospital, Hangzhou Institute of Medicine (HIM), Chinese Academy of Sciences, Hangzhou, Zhejiang 310022, China; Department of Diagnostic Ultrasound Imaging & Interventional Therapy, Zhejiang Cancer Hospital, Hangzhou Institute of Medicine (HIM), Chinese Academy of Sciences, Hangzhou, Zhejiang 310022, China; Department of Diagnostic Ultrasound Imaging & Interventional Therapy, Zhejiang Cancer Hospital, Hangzhou Institute of Medicine (HIM), Chinese Academy of Sciences, Hangzhou, Zhejiang 310022, China; Department of Diagnostic Ultrasound Imaging & Interventional Therapy, Zhejiang Cancer Hospital, Hangzhou Institute of Medicine (HIM), Chinese Academy of Sciences, Hangzhou, Zhejiang 310022, China; Interventional Medicine and Engineering Research Center, Hangzhou Institute of Medicine (HIM), Chinese Academy of Sciences, Hangzhou, Zhejiang 310022, China; Wenling Medical Big Data and Artificial Intelligence Research Institute, Taizhou, Zhejiang 317502, China; Zhejiang Provincial Research Center for Innovative Technology and Equipment in Interventional Oncology, Zhejiang Cancer Hospital, Hangzhou, Zhejiang 310022, China; Department of Diagnostic Ultrasound Imaging & Interventional Therapy, Zhejiang Cancer Hospital, Hangzhou Institute of Medicine (HIM), Chinese Academy of Sciences, Hangzhou, Zhejiang 310022, China; Department of Ultrasound, Taizhou Cancer Hospital, Taizhou, Zhejiang 317502, China; Interventional Medicine and Engineering Research Center, Hangzhou Institute of Medicine (HIM), Chinese Academy of Sciences, Hangzhou, Zhejiang 310022, China; Wenling Medical Big Data and Artificial Intelligence Research Institute, Taizhou, Zhejiang 317502, China; Department of Diagnostic Ultrasound Imaging & Interventional Therapy, Zhejiang Cancer Hospital, Hangzhou Institute of Medicine (HIM), Chinese Academy of Sciences, Hangzhou, Zhejiang 310022, China; Department of Diagnostic Ultrasound Imaging & Interventional Therapy, Zhejiang Cancer Hospital, Hangzhou Institute of Medicine (HIM), Chinese Academy of Sciences, Hangzhou, Zhejiang 310022, China; Zhejiang Provincial Research Center for Innovative Technology and Equipment in Interventional Oncology, Zhejiang Cancer Hospital, Hangzhou, Zhejiang 310022, China; Department of Diagnostic Ultrasound Imaging & Interventional Therapy, Zhejiang Cancer Hospital, Hangzhou Institute of Medicine (HIM), Chinese Academy of Sciences, Hangzhou, Zhejiang 310022, China; Zhejiang Provincial Research Center for Innovative Technology and Equipment in Interventional Oncology, Zhejiang Cancer Hospital, Hangzhou, Zhejiang 310022, China; Department of Diagnostic Ultrasound Imaging & Interventional Therapy, Zhejiang Cancer Hospital, Hangzhou Institute of Medicine (HIM), Chinese Academy of Sciences, Hangzhou, Zhejiang 310022, China; Interventional Medicine and Engineering Research Center, Hangzhou Institute of Medicine (HIM), Chinese Academy of Sciences, Hangzhou, Zhejiang 310022, China; Wenling Medical Big Data and Artificial Intelligence Research Institute, Taizhou, Zhejiang 317502, China; Zhejiang Provincial Research Center for Innovative Technology and Equipment in Interventional Oncology, Zhejiang Cancer Hospital, Hangzhou, Zhejiang 310022, China; Department of Diagnostic Ultrasound Imaging & Interventional Therapy, Zhejiang Cancer Hospital, Hangzhou Institute of Medicine (HIM), Chinese Academy of Sciences, Hangzhou, Zhejiang 310022, China

**Keywords:** medullary thyroid carcinoma, multifocality, lymph node metastasis, progression-free survival, overall survival

## Abstract

**Background:**

The prognostic role of multifocality in medullary thyroid carcinoma (MTC) is controversial. This study evaluated multifocality’s association with aggressiveness, lymph node metastasis (LNM), and survival, focusing on multifocality-related parameters (such as number of tumor foci, tumor diameter).

**Methods:**

We retrospectively analyzed 186 MTC cases (136 unifocal, 50 multifocal) with a median 59-month follow-up (95% CI: 52-66). Multivariate logistic analysis and Cox regression models assessed multifocality’s impact on LNM and recurrence, with detailed subgroup analyses. Diagnostic performance of tumor size parameters was evaluated using receiver operating characteristic (ROC) analysis, while survival outcomes were assessed via Kaplan–Meier method.

**Results:**

Multifocal tumors exhibited significantly more aggressive features, including: (1) higher preoperative calcitonin (1226.7 ± 751.9 vs 706.6 ± 704.2 ng/L, *P* < .001); (2) increased capsular invasion (68% vs 32%, odds ratio [OR] = 5.8, 95% CI: 2.32-14.53); and (3) more frequent intraglandular spread (54% vs 18%, OR = 6.05, 95% CI: 1.47-24.92). Multifocality independently predicted both LNM (OR = 3.35, 95% CI: 1.22-9.25, *P* = .019) and recurrence (hazard ratio [HR] = 6.59, 95% CI: 2.48-17.53, *P* < .001). ROC analysis identified optimal LNM cut-offs at 13.5 mm (largest focus) and 16.5 mm (total tumor diameter). Subgroup analyses revealed: (1) bifocal conferred highest LNM risk (OR = 8.51 vs unifocal, 95% CI: 1.74-41.69, *P* = .008); (2) recurrence risk showed dose–response relationship with lesion number (bifocal: HR = 4.89, 95% CI: 1.88-12.70, *P* = .001; ≥3 foci: HR = 5.86, 95% CI: 1.54-22.33, *P* = .01). Survival analysis demonstrated significantly worse progression-free survival in multifocal cases (*P* < .001), persisting beyond 2 years (*P* = .001), though overall survival difference was nonsignificant (*P* = .168).

**Conclusion:**

Multifocal MTC exhibits aggressive behavior with high LNM and recurrence risks, driven by the number of tumor foci, demonstrating a dose–response relationship. These findings support incorporating multifocality into risk stratification for optimized management.

Implications for Practice This study reveals that the number of tumor foci in medullary thyroid cancer directly correlates with recurrence risk, establishing the first dose–response relationship. Patients with multifocal tumors show significantly worse progression-free survival (*P* < .001), providing evidence to guide postoperative surveillance intervals. These findings advance precision oncology by proposing the integration of multifocality into AJCC staging and clinical decision-making, offering a practical tool to optimize individualized patient management.

## Introduction

Medullary thyroid carcinoma (MTC) is a relatively rare but aggressive type of thyroid cancer. In recent years, its incidence has shown a global upward trend. In the United States, both the incidence and mortality of MTC steadily increased between 2000 and 2019.^1^ Among patients aged 45 and older, the annual incidence rose by 1.19%.^2^ Globally, thyroid cancer ranks as the ninth most common malignancy.^3^ MTC, a neuroendocrine tumor originating from parafollicular C cells, accounts for 5%-8% of all thyroid malignancies^4^ and 15% of thyroid cancer-related deaths.^5^ Unlike differentiated thyroid carcinomas (eg, papillary and follicular carcinomas), MTC exhibits stronger invasiveness, higher rates of lymph node metastasis(LNM), and poorer prognosis, with a 10-year survival rate of approximately 75% when regional lymph nodes are involved.^6^ Distant metastases at diagnosis reduce 10-year survival significantly to 10%-40%.^7^ Therefore, investigating the clinicopathological features and prognostic factors of MTC is critical for optimizing treatment strategies and improving patient outcomes.

Multifocality is a key characteristic of thyroid cancer and has been extensively studied in papillary thyroid carcinoma (PTC). Multiple studies demonstrate that multifocal PTC is associated with higher LNM rates,^8^ increased recurrence risk,^9^ and worse prognosis.^10^ However, research on MTC multifocality remains limited, with small sample sizes and unclear clinical significance. Existing literature suggests that multifocal MTC may correlate with greater invasiveness and poorer outcomes, though evidence is conflicting.^11–13^ Furthermore, whether multifocality in MTC is linked to calcitonin levels, local invasion, LNM, or long-term survival requires further exploration.

This study aims to compare clinicopathological features, invasiveness, LNM, and survival between unifocal and multifocal MTC patients through retrospective cohort analysis. The clinical significance of multifocality and tumor number in MTC is also evaluated.

## Methods

### Study population

**Figure 1:**
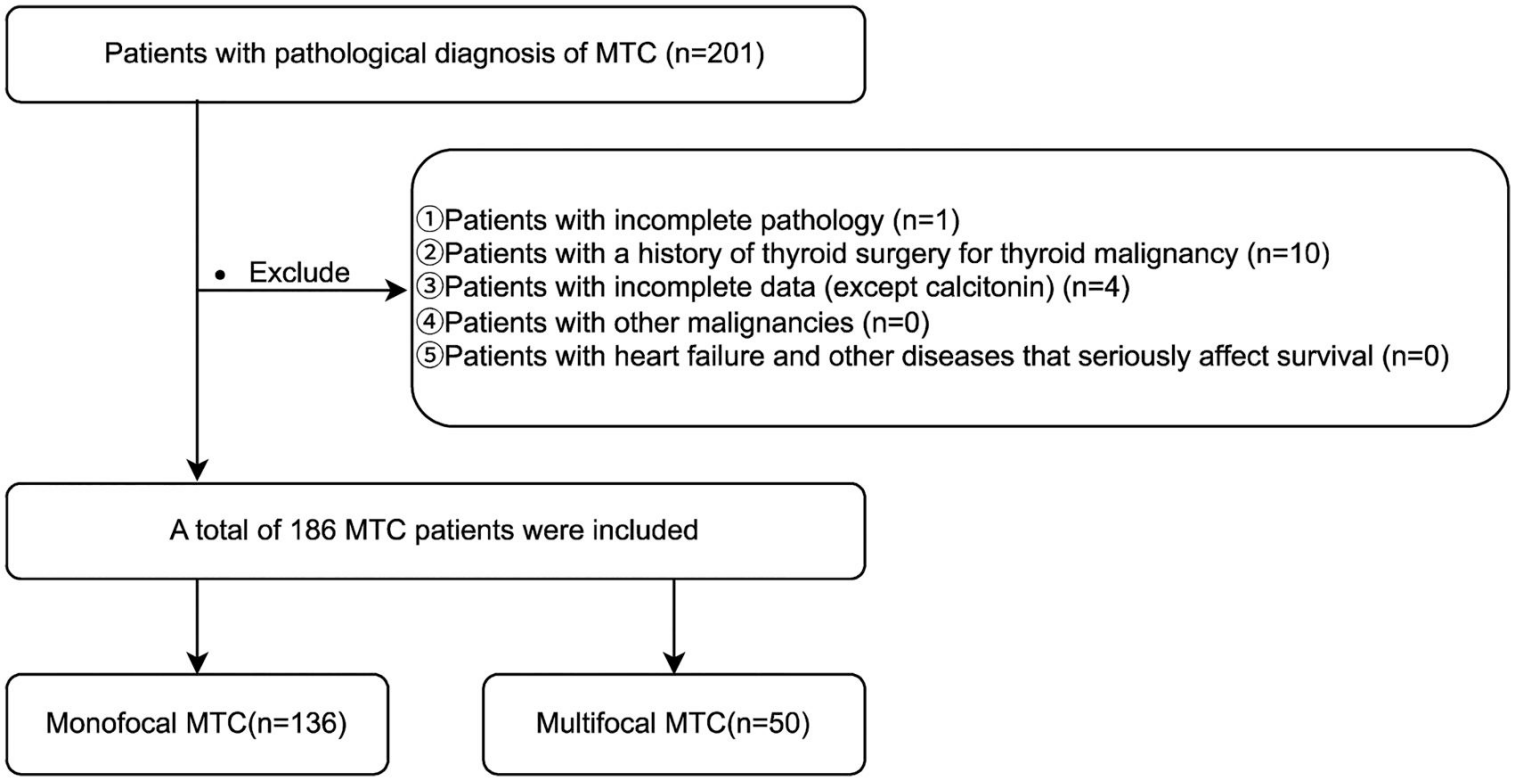
A flowchart of patient and nodule inclusion and exclusion.

A retrospective analysis was conducted on patients diagnosed with MTC at Zhejiang Cancer Hospital between January 1, 2012 and December 31, 2022. The study protocol was reviewed and approved by the Ethics Committee of Zhejiang Cancer Hospital (Approval No.IRB-2024-580, June 11, 2024) granted a waiver of informed consent for this retrospective study as it involved only analysis of anonymized existing data. All procedures complied with the Declaration of Helsinki (2013). As this was a retrospective analysis of existing clinical data with no additional interventions, the need for individual patient consent was waived by the ethics committee. All data were de-identified and analyzed anonymously. Clinical and pathological data were collected, and patients with a history of thyroid malignancy surgery (*n* = 10) or incomplete records (*n* = 4) were excluded. A total of 186 patients were enrolled, including 13 cases with concomitant PTC. Based on the final postoperative pathological findings, patients were stratified into unifocal and multifocal groups for comparative analysis of clinicopathological characteristics and long-term oncologic outcomes (Figure 1). The diagnostic criterion was histopathologically confirmed MTC. Focality was classified as unifocal (single tumor focus) or multifocal (≥2 foci, regardless of laterality).

### Data collection and follow-up

Demographic, clinicopathological, and survival data were extracted from medical records. Key variables included: Tumor characteristics: Focality (unifocal/multifocal), size, location, TNM stage (AJCC 8th ed.). Pathological markers: Capsular invasion, intraglandular spread, LNM. Biochemical data: Pre/postoperative calcitonin levels (truncated at 0/2000 ng/L). Outcomes: Recurrence, survival status, follow-up duration. The median follow-up was 59 months (95% CI: 52-66). Recurrence was defined as disease progression after surgery, including local recurrence, LNM, contralateral MTC, or distant metastasis. Progression-free survival (PFS) was calculated from surgery to recurrence or last follow-up, while overall survival (OS) was measured from treatment to death or last follow-up.

### Statistical analysis

Patients were stratified into unifocal and multifocal groups based on tumor number. Clinicopathological characteristics and survival outcomes were compared between groups. Continuous variables were analyzed using *t*-tests, and categorical variables with chi-square tests. Missing calcitonin data were handled by mean imputation. For calcitonin comparisons, analysis of covariance was used when variances were equal; otherwise, generalized linear models were applied. Multicollinearity was assessed using Pearson correlation coefficients and variance inflation factors.

Receiver operating characteristic (ROC) curves evaluated the predictive value of maximum tumor diameter and sum of diameters for LNM. Univariate and multivariate logistic analyses were performed to identify independent risk factors for LNM and recurrence, while the Cox proportional hazards model was used to assess recurrence risk. Subgroup analyses were conducted to investigate the mechanisms underlying the associations between multifocality and LNM/recurrence, including total tumor diameter, bilaterality (yes vs no), maximum diameter, and number of lesions (1 focus vs. 2 foci/≥3 foci), with multivariate logistic analysis/Cox regression models incorporated. Mean follow-up was calculated using the inverse Kaplan–Meier method, and survival curves were compared via log-rank tests. To assess the potential confounding effect of the extent of surgery on the primary outcome (recurrence), variables for total thyroidectomy, central neck dissection (CND), and lateral neck dissection (LND) were included as covariates in the multivariate Cox proportional hazards model. A *P*-value <.05 was considered statistically significant. All analyses were performed using SPSS 26.0 (IBM Corp.).

This study aimed to determine whether multifocal MTC is associated with increased tumor aggressiveness, higher LNM rates, and worse long-term outcomes compared to unifocal disease, with implications for staging and treatment strategies.

## Results

Patient characteristics A total of 186 patients were included in this study, comprising 82 males (44.1%) and 104 females (55.9%), with a median age of 49.7 years (IQR: 40-59). Among them, 136 patients (73.1%) had unifocal MTC, while 50 (26.9%) exhibited multifocal disease, including 34 (18.3%) with bilateral involvement. The median maximum tumor diameter was 20 mm (IQR: 10-26), with 52 cases (28%) classified as microcarcinomas (≤10 mm). Pathological examination revealed capsular invasion in 83 patients (44.6%), intraglandular spread in 19 (10.2%), and vascular invasion in 24 (12.9%). Lymph node metastases were identified in 109 cases (58.6%), including 33 (17.7%) with N1a disease and 83 (18.6%) with N1b involvement. Distant metastases (M1) were present in 7 patients (3.8%), predominantly affecting the lungs (*n* = 5, 71.4%), with 2 cases (28.6%) demonstrating metastases at 2 anatomical sites. The mean follow-up duration, calculated using the reverse Kaplan–Meier method to account for high censoring, was 59 months (95% CI: 52-66). During follow-up, 30 patients (16.1%) experienced recurrence, with 8 MTC-related deaths and 2 non-MTC deaths recorded. Missing calcitonin values (preoperative: 13%; postoperative day 7: 4%; month 3: 12%) were imputed using mean substitution to maximize sample retention.

Comparative analysis demonstrated significant differences between unifocal and multifocal groups. Multifocal cases were younger (43.9 ± 15.5 vs 51.9 ± 12.8 years, *P *< .01) and exhibited higher preoperative calcitonin levels (1226.7 ± 751.9 vs 706.6 ± 704.2 ng/L, *P *< .001), increased familial predisposition (*P *< .001), more frequent capsular invasion (*P *< .001), intraglandular spread (*P *< .001), LNM (*P *< .001), and advanced TNM staging (*P *< .01). No significant difference was observed in concomitant PTC incidence between groups (Table 1). Among patients with preoperative calcitonin <200 pg/mL (*n* = 57), those with multifocal disease (*n* = 9) had a 2.8-fold higher prevalence of AJCC stage III-IV disease compared to those with unifocal disease (55.5% vs 35.4%; OR = 2.8).

**Table 1:** Baseline clinicopathological characteristics of patients.

Variable	Total (*n* = 186)	Unifocality	Multifocality	*P*
		(*n* = 136, 73.1%)	(*n* = 50, 26.9%)	
**Gender (male), *n* (%)**	82 (44.1%)	56 (41.2%)	26 (52%)	.187
**Age (y)**	49.7 ± 14.0 (IQR 40-59)	51.9 ± 12.8	43.9 ± 15.5	**.001**
**Family history**	17 (9.1%)	6 (4.4%)	11 (22%)	**<.001**
**Side, *n* (%)**				
** Left**	72 (38.7%)	66 (48.5%)	6 (12%)	**<.001**
** Right**	80 (43.0%)	70 (51.5%)	10 (20%)	
** Bilaterality**	34 (18.3%)	0	34 (68%)	
**Concurrent PTC**	13 (7.0%)	12 (8.8%)	1 (2%)	.106
**Pre-operative calcitonin (ng/L)**	846.4 ± 751.7	706.6 ± 704.2	1226.7 ± 751.9	**<.001**
**Calcitonin (ng/L), POD7**	162.1 ± 381.7	96.0 ± 259.4	341.9 ± 565.3	**.008**
**Calcitonin (ng/L), POM3**	150.3 ± 395.9	83.1 ± 271.7	333.1 ± 584.5	**.008**
**Surgical characteristic**				
**Total thyroidectomy, *n* (%)**	129 (69.4%)	88 (64.7%)	41 (82%)	**.023**
**Central neck dissection, *n* (%)**	171 (91.9%)	122 (89.7%)	49 (98%)	.066
**Lateral neck dissection, *n* (%)**	90 (48.4%)	59 (43.4%)	31 (62%)	**.024**
**Tumor foci**				
** 1**	136 (73.1%)			
** 2**	32 (17.2%)			
** 3**	11 (5.9%)			
** ≥4**	7 (3.8%)			
**Maximum diameter (mm)**	20.0 ± 13.5 (IQR 10.0-26.0)	18.9 ± 13.1	23.1 ± 14.4	.061
** ≤1 cm**	52 (28.0%)	42 (31%)	10 (20%)	
** >1 cm**	134 (72.0%)	94 (69%)	40 (80%)	
**Total tumor diameter (mm)**	23.8 ± 18.1 (IQR 10.8-33.0)	18.9 ± 13.1	37.2 ± 22.8	**.001**
**Capsular invasion**	83 (44.6%)	50 (36.8%)	33 (66%)	**<.001**
**Intrathyroidal dissemination**	19 (10.2%)	7 (5.1%)	12 (24%)	**<.001**
**Lymphovascular invasion**	24 (12.9%)	13 (9.6%)	11 (22%)	**.025**
**Associated Hashimoto’s thyroiditis**	18 (9.7%)	14 (10.3%)	4 (8%)	.639
**Calcification**	14 (7.5%)	9 (6.6%)	5 (10%)	.438
**Collagenization**	13 (7.0%)	10 (7.4%)	3 (6%)	.748
**T Stage**				
** T1**	104 (55.9%)	85 (62.5%)	19 (38%)	**.003**
** T2**	47 (25.3%)	31 (22.8%)	16 (32%)	
** T3**	18 (9.7%)	13 (9.6%)	5 (10%)	
** T4**	17 (9.3%)	7 (5.1%)	10 (20%)	
**N stage**				
** N0**	70 (37.6%)	63 (46.3%)	7 (14%)	**<.001**
** N1aCLNM**	33 (17.7%)	19 (14.0%)	14 (28%)	
** N1bLLNM**	83 (44.6%)	54 (39.7%)	29 (58%)	
**Lymphatic metastasis**	116 (62.4%)	73 (53.7%)	43 (86%)	**<.001**
**M1, *n* (%)**	7 (3.8%)	4 (2.9%)	3 (6%)	.331
**AJCC, *n* (%)**				
** I**	54 (29%.0)	48 (35.3%)	6 (12%)	**.001**
** II**	15 (8.1%)	14 (10.3%)	1 (2%)	
** III**	33 (17.7%)	19 (14.0%)	14 (28%)	
** IV**	84 (45.2%)	55 (40.4%)	29 (58%)	
**Follow-up (y)**	4.33 ± 3.53 (IQR 1.09-6.53)	4.28 ± 3.51	4.49 ± 3.59	.715
**Recurrence**	30 (16.1%)	12 (8.8%)	18 (36%)	**<.001**
**Mortality**	8 (5.4%)	5 (3.7%)	5 (10%)	.123

Abbreviations: AJCC, American Joint Committee on Cancer; POD7, postoperative day 7; POM3, postoperative month 3; calcitonin reference range: 0.00–8.40 ng/L; Bold values indicate statistically significant differences (*P* < 0.05).

### Impact of multifocality on LNM

**Figure 2:**
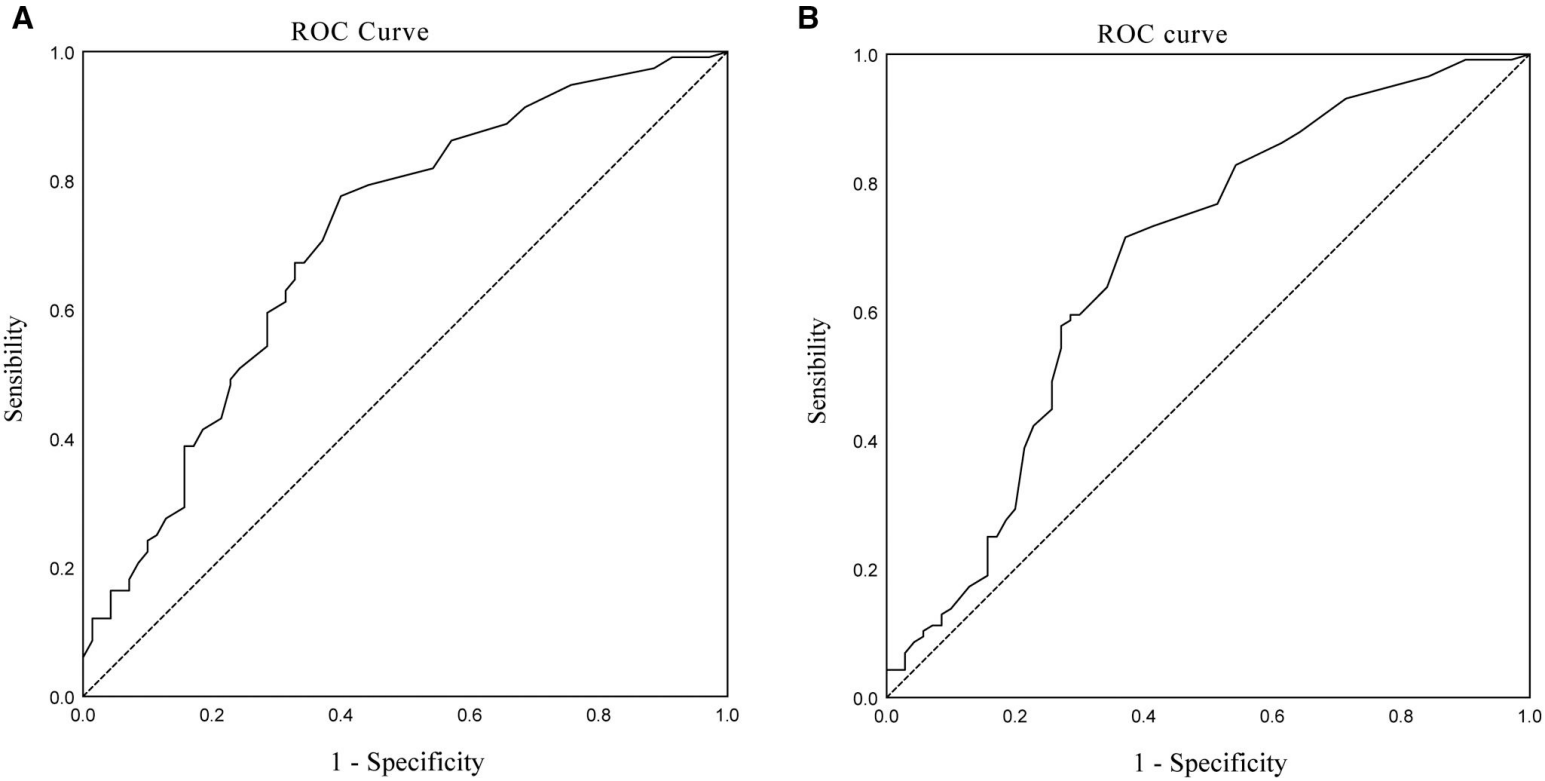
The ROC curve of the maximum diameter and TTD for predicting LNM. (A) The ROC curve of the maximum diameter and (B) the ROC curve of TTD. Abbreviations: LNM, lymph node metastasis; ROC, receiver operator characteristic; TTD, total tumor diameter.

Multivariate analysis identified multifocality as an independent predictor of LNM (odds ratio [OR] = 3.35, 95% CI : 1.22-9.25, *P *= .019), second only to capsular invasion (OR = 5.8, 95% CI: 2.32-14.53, *P *< .001) (Table 2). Tables S1-S3 summarize the collinearity diagnostics and correlation matrices for covariates in the multivariable regression models. ROC analysis (Figure 2) demonstrated that the optimal cutoff value was 13.5 mm for maximum diameter (AUC = 0.71, sensitivity 77.6%, specificity 60%) and 16.5 mm for summed diameters (AUC = 0.68, sensitivity 59.5%, specificity 71.4%). To further investigate the mechanisms underlying multifocality’s effects, subgroup models were constructed by pairing “total tumor diameter and bilaterality” with “maximum diameter and number of tumor foci” based on clinical relevance and collinearity (Table 3). Subgroup analyses revealed that neither total tumor diameter, bilaterality, nor maximum tumor size demonstrated statistical significance. However, when using unifocal cases (Foci1) as the reference group, bifocal cases (Foci2) showed significantly increased LNM risk (OR = 8.512, 95% CI: 1.738-41.693, *P *= .008), while ≥3 lesions (Foci ≥ 3) had no significant impact (OR = 1.152, 95% CI: 0.271-4.902, *P *= .848). The sample size for the ≥3 lesions (Foci ≥ 3) dummy variable was 18. Fisher’s exact test indicated significant differences in LNM proportions across focality groups (*P *< .001), with the bifocal group exhibiting higher LNM incidence (93.8%) than both unifocal (53.7%) and ≥3 lesions groups (72.2%).

**Table 2:** Logistic analysis of risk factors for lymphatic metastasis.

	Univariate analysis				Multivariate analysis				
		95% CI				95% CI			Cut-off value
Variable	OR	lower	upper	*P*	OR	lower	upper	*P*	
**Gender（male）**	0.58	0.31	1.06	.075					
**Age (y)**	0.99	0.97	1.01	.385					
**Family history**	2.08	0.65	6.66	.216					
**Concurrent PTC**	0.69	0.22	2.13	.513					
**Pre-operative calcitonin (ng/L)**	1.00	1.00	1.00	**<.001**	1.00	1.00	1.00	.065	
**Multifocality**	5.30	2.23	12.62	**<.001**	3.35	1.22	9.25	**.019**	
**Tumor foci**									
** 1**	Reference	–	–	**.001**					
** 2**	12.95	2.98	56.33	**.001**					
** ≥3**	2.24	0.76	6.64	.144					
**Maximum diameter (mm)**	1.04	1.02	1.07	**.002**					13.50
**Total tumor diameter (mm)**	1.05	1.02	1.08	**<.001**					16.50
**Bilaterality**	3.39	1.33	8.68	**.011**					
**Capsular invasion**	11.94	5.39	26.46	**<.001**	5.80	2.32	14.53	**.000**	
**Intrathyroidal dissemination**	12.67	1.65	97.18	**.015**	3.18	0.31	32.19	.328	
**Lymphovascular invasion**	17.07	2.25	129.44	**.006**	5.32	0.55	51.33	.149	
**Associated Hashimoto’s thyroiditis**	0.73	0.27	1.95	.532					
**T stage**									
** T1**	Reference	–	–	**.000**	Reference	–	–		
** T2**	2.82	1.34	5.96	**.006**	1.37	0.49	3.78	.547	
** T3-4**	11.52	3.32	39.98	**.000**	1.94	0.43	8.87	.391	
**M Stage**	3.76	0.44	31.93	.224					

Abbreviations: OR, odds ratio; Bold values indicate statistically significant differences (*P* < 0.05).

**Table 3:** Subgroup analysis of the impact of multifocality on LNM and recurrence of MTC.

Model_Type	Variable	*P*	OR	Lower_CI	Upper_CI
**LNM_Logistic_1**	Total tumor diameter	.498	0.985	0.944	1.028
**LNM_Logistic_1**	Bilaterality	.118	2.691	0.778	9.299
**LNM_Logistic_2**	Maximum diameter	.332	0.975	0.925	1.027
**LNM_Logistic_2**	Tumor Foci_Foci1 (Reference)	.031	1	1	1
**LNM_Logistic_2**	Tumor Foci_Foci2	.008	8.512	1.738	41.693
**LNM_Logistic_2**	Tumor Foci_Foci ≥3	.848	1.152	0.271	4.902
**Recurrence_Logistic_1**	Bilaterality	.173	2.826	0.635	12.573
**Recurrence_Logistic_1**	Total tumor diameter	.512	1.013	0.976	1.051
**Recurrence_Logistic_2**	Maximum diameter	.244	1.028	0.981	1.077
**Recurrence_Logistic_2**	Tumor Foci_Foci1 (Reference)	.018	1	1	1
**Recurrence_Logistic_2**	Tumor Foci_Foci2	.026	4.079	1.187	14.022
**Recurrence_Logistic_2**	Tumor Foci_Foci ≥3	.023	6.105	1.282	29.084

Model_Type	Variable	*P*	HR	Lower_CI	Upper_CI
**Recurrence_Cox_1**	Bilaterality	.249	2.015	0.613	6.63
**Recurrence_Cox_1**	Total tumor diameter	.347	1.012	0.987	1.039
**Recurrence_Cox_2**	Maximum diameter	.051	1.031	1	1.063
**Recurrence_Cox_2**	Tumor Foci_Foci1 (Reference)	.002	1	1	1
**Recurrence_Cox_2**	Tumor Foci_Foci2	.001	4.891	1.883	12.701
**Recurrence_Cox_2**	Tumor Foci_Foci ≥3	.01	5.859	1.537	22.334

Abbreviations: HR, hazard ratio; OR, odds ratio.

Impact on Recurrence Risk (Table 4) Multifocality was independently associated with significantly increased recurrence risk in MTC, as demonstrated by 2 distinct analytical approaches. Multivariate logistic regression analysis revealed that multifocal tumors had a 5.05-fold higher recurrence risk than unifocal lesions (OR = 5.05, 95% CI: 1.67-15.22, *P *= .004). The Cox proportional hazards model demonstrated an even greater association, with a 6.59-fold increased recurrence risk (hazard ratio [HR] = 6.59, 95% CI: 2.48-17.53, *P *< .001). The recurrence risk was particularly enhanced when multifocality coexisted with other pathological features, including intraglandular spread (HR = 6.05, 95% CI: 1.47-24.92, *P *= .013) and maximum tumor diameter exceeding 1 cm (HR = 4.16, 95% CI: 1.11-15.53, *P *= .034). To address the potential confounding effect of family history, a sensitivity multivariate analysis was performed. After adjustment for family history, multifocality persisted as a strong predictor of lymph node metastasis (adjusted OR = 2.80, 95% CI: 0.98-8.01, *P* = .054). For the endpoint of recurrence, multifocality remained a significant independent risk factor after adjustment (adjusted HR = 5.07, 95% CI: 1.59-16.15, *P *= .006). Tables S4-S6 summarize the collinearity diagnostics and correlation matrices for covariates in the multivariable regression models. Subgroup analyses revealed that in the Cox proportional hazards model (Table 3): Compared with unifocal lesions, bifocal lesions (Foci2) showed significantly increased recurrence risk (HR = 4.891, 95% CI: 1.883-12.701, *P *= .001), while lesions with ≥3 foci (Foci ≥ 3) exhibited further elevated risk (HR = 5.859, 95% CI: 1.537-22.334, *P *= .01). Bilaterality demonstrated a higher-risk trend (HR = 2.015, 95% CI: 0.987-6.63) that did not reach statistical significance (*P *= .249). A similar pattern was observed in the logistic regression model: bifocal lesions had an OR = 4.079 (95% CI: 1.187-14.022, *P *= .026), lesions with ≥3 foci had an OR = 6.105 (95% CI: 1.282-29.084, *P *= .023), and bilaterality showed an OR = 2.826 (95% CI: 0.635-12.573, *P *= .173). The presence of concomitant PTC was not significantly associated with multifocality, lymph node metastasis, or disease recurrence in univariate analysis (all *P *> .1), and was therefore not considered a primary confounder in subsequent multivariate models.

**Table 4:** Analysis of independent risk factors for MTC recurrence.

	Univariate analysis				Multivariate logistic analysis				Multivariate Cox regression analysis			
		95% CI				95% CI				95% CI		
Variable	OR	lower	Upper	*P*	OR	lower	upper	*P*	HR	lower	upper	*P*
**Gender（male）**	0.64	0.29	1.41	.268								
**Age (y)**	0.98	0.95	1.01	.112								
**Family history**	1.69	0.51	5.60	.389								
**Concurrent PTC**	0.94	0.20	4.48	.940								
**Pre-operative calcitonin (ng/L)**	1.00	1.00	1.00	**<.001**	1.00	1.00	1.00	.320	1.00	1.00	1.00	.064
**Calcitonin (ng/L), POD7**	1.00	1.00	1.00	**<.001**	1.00	1.00	1.00	.702	1.00	1.00	1.00	.669
**Calcitonin (ng/L), POM3**	1.00	1.00	1.00	**<.001**	1.00	1.00	1.00	.229	1.00	1.00	1.00	.913
**Multifocality**	5.81	2.54	13.30	**<.001**	5.05	1.67	15.22	**.004**	6.59	2.48	17.53	**.000**
**Tumor foci**												
** 1**	Reference	–	–	**<.001**								
** 2**	5.41	2.12	13.85	**<.001**								
** ≥3**	6.58	2.15	20.11	**.001**								
**Maximum diameter (mm)**	1.04	1.01	1.07	**.003**								
** >1 cm**	2.89	0.96	8.73	.060	2.78	0.60	12.77	.189	4.16	1.11	15.53	**.034**
**Total tumor diameter (mm)**	1.04	1.02	1.07	**<.001**								
**Bilaterality**	4.06	1.72	9.58	**.001**								
**Capsular invasion**	5.26	2.13	13.00	**<.001**	0.90	0.22	3.72	.890	1.20	0.34	4.25	.774
**Intrathyroidal dissemination**	8.17	2.96	22.52	**<.001**	6.05	1.47	24.92	**.013**	1.55	0.57	4.21	.392
**Lymphovascular invasion**	5.07	1.99	12.94	**.001**	1.18	0.29	4.73	.815	1.83	0.57	5.86	.312
**Associated Hashimoto’s thyroiditis**	2.20	0.72	6.71	.166								
**T stage**												
** T1**	Reference	–	–	**.003**	Reference	–	–					
** T2**	1.24	0.43	3.57	.694	0.17	0.03	0.81	**.026**	0.14	0.04	0.53	**.004**
** T3**	3.25	0.97	10.86	.055	0.50	0.09	2.89	.438	0.35	0.09	1.29	.116
** T4**	7.52	2.41	23.48	**.001**	0.40	0.07	2.40	.315	0.24	0.06	1.04	.057
**N stage**												
** N0**	Reference	–	–	**<.001**	Reference	–	–					
** N1aCLNM**	0.70	0.07	6.98	.759	0.17	0.01	2.24	.177	0.13	0.01	1.49	.102
** N1bLLNM**	10.19	2.93	35.42	**<.001**	4.29	0.84	21.85	.079	4.12	0.90	18.90	.068
**Lymphatic metastasis**	6.78	1.97	23.28	**.002**								
**M1**	0.86	0.10	7.43	.893								
**AJCC**												
** I**	Reference			**<.001**								
** II**	1.86	0.16	22.00	.624								
** III**	0.81	0.07	9.33	.868								
** IV**	11.66	2.64	51.52	**.001**								

Abbreviations: HR, hazard ratio; OR, odds ratio; Bold values indicate statistically significant differences (*P* < 0.05).

CEA data were available for 123 patients (66.1%). Its levels were not associated with multifocality, lymph node metastasis, or disease recurrence in univariate analyses (all *P *> .6). An analysis of missing data did not reveal significant bias. Therefore, CEA was not considered in the multivariate prognostic models.

### Association of multifocality with surgical extent and prognosis

Patients with multifocal disease were more likely to undergo total thyroidectomy and lateral neck dissection (Table 1). In a logistic regression model, multifocality was independently associated with the performance of LND (OR = 2.13, 95% CI 1.10-4.12, *P* = .026). Crucially, in the multivariate Cox regression model for recurrence—which included these surgical variables (total thyroidectomy, CND, LND) and M1 status as covariates—multifocality remained a strong and independent predictor (adjusted HR= 6.96, 95% CI 2.56-18.90, *P* < .001), whereas none of the surgical variables were significant in this model. Among the 30 patients with disease recurrence, detailed imaging data to localize the site of structural progression were available for 26 patients. The distribution of recurrence sites in these 26 patients was as follows: lateral neck only in 14 patients (53.8%), central neck only in 4 patients (15.4%), both central and lateral neck in 3 patients (11.5%), and distant metastasis in 5 patients (19.2%).

### Survival outcomes

**Figure 3:**
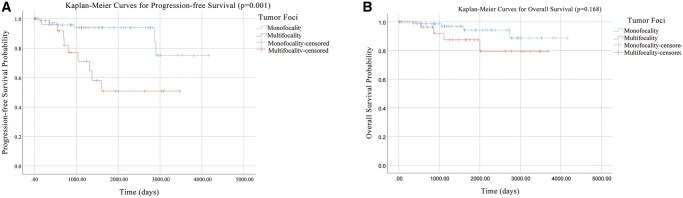
Progression-free survival and overall survival curves at 2-year follow-up.

Kaplan–Meier analysis demonstrated significantly worse PFS in multifocal cases (*P *< .001). When early events within the first 2 years were excluded, the PFS difference remained statistically significant in the follow-up period beyond 2 years (*P *= .001). The median recurrence time was not reached in the unifocal group as over 50% of patients remained event-free. No significant difference in OS was observed (*P *= .168), with only 8 MTC-related deaths recorded during follow-up. Although the OS curves showed a separation trend beyond 2 years, this difference did not reach statistical significance (*P *= .168, Figure 3).

## Discussion

This study retrospectively analyzed clinical data from 186 MTC patients in a single-center cohort to investigate the clinical significance of multifocality in MTC and its impact on LNM and recurrence. This represents the largest single-center study on survival outcomes in multifocal medullary thyroid carcinoma to date.^14^ The results demonstrate that multifocal MTC is associated with distinct clinicopathological characteristics, including younger age at diagnosis, significantly higher calcitonin levels, increased rates of capsular invasion and intraglandular spread, greater LNM, and more advanced AJCC staging—all indicative of a more aggressive tumor phenotype. The association between multifocality and tumor aggressiveness has been similarly demonstrated in studies of PTC.^9^^,^^10^ Of particular interest is the observed 7% incidence of concomitant PTC in our cohort, a remarkably high co-occurrence rate.^15^ This phenomenon may potentially be explained by either: (1) shared tumorigenic stimuli leading to concurrent transformation of different thyroid cell types^16^ or (2) common genetic origins as suggested by emerging evidence.^17^ Although in our cohort concomitant PTC was not associated with multifocality or with the primary endpoints of lymph node metastasis or recurrence in univariate analysis (both *P* > .5), and therefore unlikely to be a major confounder, its presence could theoretically influence the assessment of disease-specific behavior. Future studies with larger cohorts may benefit from stratified analyses to further validate the independent prognostic role of MTC multifocality in the absence of concurrent pathologies.

This study identifies multifocality and capsular invasion as dominant independent risk factors for LNM in MTC (Table 2). Multifocality was associated with a 3.35-fold higher odds of LNM (OR = 3.35, 95% CI: 1.22-9.25, *P *= .019), corroborating Andreas Machens’ findings of multifocal growth correlating with nodal metastasis (OR = 2.5, *P *= .01).^13^ Multifocality also serves as a significant risk factor for lateral cervical LNM.^18^ In PTC, multifocal lesions typically originate from a single malignant clone, with clonal progression and morphological heterogeneity arising through subclonal evolution following intraglandular spread.^19^ In contrast, MTC may develop LNM via multicentric independent tumor foci or clonal dissemination. Clinically actionable size thresholds were established (maximum diameter >13.5 mm; summed diameters >16.5 mm) for LNM prediction, aligning with PTC data where tumor size (≥1cm) independently predicts nodal metastasis (pooled OR = 3.53).^20^ These metrics may guide compartment-oriented lymphadenectomy decisions even in radiologically node-negative cases. Subgroup analyses suggested that bifocal lesions (Foci2) had significantly higher LNM risk compared to unifocal lesions, while lesions with ≥3 foci did not exhibit further increased LNM risk, potentially due to sample size limitations or tumor biological heterogeneity.

Multifocality was identified as the most robust independent predictor of recurrence in MTC, demonstrating in logistic analysis and the Cox proportional hazards model (Table 4). The effect sizes exceed previously reported values,^13^^,^^21^^,^^22^ suggesting that multifocality in MTC may not merely reflect tumor burden but could indicate either: (1) the presence of occult micrometastases directly driving recurrence or (2) a regional field cancerization of C cells^23^ Intraglandular spread was another independent predictor, associated with a 6.05-fold increased recurrence risk (OR = 6.05, 95% CI: 1.47-24.92, *P *= .013), consistent with histological evidence that tumor satellite foci within thyroid parenchyma may evade surgical eradication.^24^ The robust association between multifocality and recurrence (adjusted HR = 5.07, *P* = .006), even after adjusting for family history, solidifies its role as a key prognostic indicator. Subgroup analyses indicated that only the number of tumor foci was an independent risk factor for recurrence, with risk escalating in a dose-dependent manner. Patients with ≥3 tumor foci demonstrated significantly increased recurrence risk, with an OR of 6.105 (95% CI: 1.282-29.084, *P *= .023) in logistic regression analysis and a HR of 5.859 (95% CI: 1.537-22.334, *P *= .01) in Cox proportional hazards analysis. This suggests that recurrence risk in MTC is primarily determined by the absolute number of tumor foci rather than maximum diameter or total tumor diameter. These findings may integrate distinct mechanisms in sporadic and hereditary MTC: in sporadic MTC, multifocality reflects clonal dissemination via lymphatic spread, whereas hereditary MTC arises from multicentric malignant transformation.^25^ Despite differing origins, the focus count comprehensively reflects overall tumor aggressiveness, supporting its role as a universal prognostic indicator. Future studies should validate this hypothesis through molecular subtyping (eg, RET/RAS mutation profiling). Although bilaterality did not reach statistical significance (*P *> .05), its HR (2.015) and OR (2.826) suggest a clinically relevant trend warranting larger-scale validation. The minimal effect size of maximum diameter (HR = 1.031, *P *= .051) underscores the superior predictive value of lesion count; prophylactic lateral neck dissection should be considered for tumors with ≥3 lesions, even if maximum diameter is <2 cm.^26^ However, tumors with maximum diameter >1 cm showed a 3.16-fold increased recurrence risk (HR = 4.16, 95% CI: 1.11-15.53, *P = *.034), aligning with the established prognostic framework of MTC and supporting the biological rationale for the T1a/T1b distinction in AJCC staging.^27^ This size-dependent risk progression likely reflects enhanced angiogenic potential and invasive capacity.^28^ Furthermore, our analysis of surgical extent revealed that multifocality was associated with more comprehensive initial surgery, yet it remained an independent risk factor for recurrence after adjusting for these surgical parameters. This indicates that the adverse prognosis linked to the multifocal phenotype is likely driven by its inherent biological aggressiveness rather than by differences in surgical management. These findings reinforce the potential value of preoperative identification of multifocality in guiding the initial surgical strategy toward a more comprehensive resection.

Kaplan–Meier analysis robustly demonstrated that PFS was significantly worse in multifocal MTC patients compared to unifocal cases (*P *= .001). This difference remained statistically significant even after exclusion of early events within the first 2 years, indicating multifocality as a persistent driver of recurrence risk. Although OS curves showed a nonsignificant trend toward separation (OR = 0.168), this aligns with prior observations that MTC-related mortality often lags behind recurrence by years,^29^ and high local recurrence rates do not directly impact OS due to effective structural disease control through reoperations.^30^ The divergence between PFS and OS underscores the clinical relevance of recurrence as an actionable endpoint, particularly in multifocal cases where early biochemical or structural relapse may guide timely intervention. Further long-term follow-up studies are warranted, given the potential for late recurrences decades after initial treatment.^31^ This finding carries a direct implication for preoperative management. Although ultrasound diagnosis of multifocal MTC can be challenging due to its often atypical appearance, our data establish that the presence of multifocality itself is a paramount risk factor. Consequently, when multifocality is identified or strongly suspected preoperatively—prompted by a thorough ultrasound evaluation and a prudent approach to biopsying multiple nodules—it should serve as a decisive indicator for considering an initial surgical strategy that addresses its high-risk nature, such as total thyroidectomy with central neck dissection. This approach is justified by the significant association we have demonstrated between multifocality and aggressive disease behavior.

Persistently elevated calcitonin levels were observed in multifocal MTC patients both preoperatively and post-thyroidectomy, indicating greater tumor burden and biological aggressiveness compared to unifocal cases.^32^ While preoperative calcitonin reflects quantitative secretory burden, multifocality captures a qualitative, spatially aggressive phenotype. The superior and independent prognostic power of multifocality for metastasis and recurrence was established in our multivariate models. Its ability to identify high-risk patients even with sub-200 pg/mL calcitonin highlights its distinct utility in preoperative planning. Early nodal recurrence requiring reoperation within one year was associated with incomplete calcitonin normalization, suggesting occult residual disease.^33^ While secondary surgery achieved biochemical remission in select cases, conventional imaging demonstrated limited sensitivity for detecting residual lesions, particularly at calcitonin levels <500 pg/mL.^34^ These findings support consideration of extended initial surgery and rigorous postoperative surveillance in multifocal MTC, while highlighting the need for improved detection methods and molecular characterization of multifocal disease progression.^35^^,^^36^

This single-center retrospective study, while representing the largest cohort investigating multifocality’s impact on MTC survival, has several limitations. Selection bias may exist due to the retrospective design. Genetic testing was not routinely performed, preventing definitive stratification between hereditary and sporadic MTC cases. Consequently, we could not determine whether the observed multifocal phenotype serves as a surrogate for specific genetic drivers, such as RET wild-type status or RAS mutations. While the low familial incidence (9.1%) suggests a limited overall impact of hereditary cases, the integration of comprehensive genetic profiling in future studies is essential. Such research will be crucial to elucidate the molecular mechanisms underlying multifocality and to validate its independent prognostic significance against defined genetic alterations. Since multifocal cases tended to receive more extensive resections, the observed association between multifocality and recurrence might partially reflect residual confounding. Propensity score-matched analyses are warranted to verify multifocality’s independent prognostic effect. Follow-up duration was insufficient to capture late events, precluding median PFS/OS estimation. Despite these limitations, multifocality was consistently identified as an independent recurrence risk factor. Multicenter prospective studies with extended follow-up are needed to validate these findings.

## Conclusions

Multifocality serves as a critical marker of aggressiveness in MTC, functioning as an independent predictor of higher LNM rates and recurrence risks. Its risk stratification value is primarily driven by the number of tumor foci, demonstrating a dose–response relationship. The persistent impact of multifocality on progression-free survival confirms its role as a long-term prognostic factor. Although no statistically significant difference in overall survival was observed (Log-rank *P *= .168), likely due to therapeutic effects or follow-up limitations, the elevated recurrence risk in multifocal cases necessitates more comprehensive initial treatment strategies and intensified surveillance protocols. Surgical decision-making should prioritize focus count, while surveillance strategies require stratification by focus number.

These findings emphasize the clinical relevance of multifocality in MTC management and highlight the need for further investigation into its underlying biological mechanisms. Future prospective studies should focus on validating these observations and (1) determining tumor number thresholds and their interaction with family history; (2) developing risk stratification models integrating multifocality, molecular markers, and genetic background; and (3) investigating the biological mechanisms driving multifocality. These studies will refine precision medicine approaches for MTC management.

## Supplementary Material

oyag065_Supplementary_Data

## Data Availability

The data underlying this article will be shared on reasonable request to the corresponding author.
