# Expression of Concern: Multifocality as a marker of aggressiveness in medullary thyroid carcinoma: a retrospective cohort analysis of lymph node metastasis and recurrence

**DOI:** 10.1093/oncolo/oyag383

**Published:** 2026-10-19

This is an Expression of Concern regarding: Yidan Lu, Ziyi Chen, Jing Yang, Tian Jiang, Na Feng, Jincao Yao, Di Ou, Zhiyan Jin, Liyu Chen, Chen Yang, Dong Xu, Lingyan Zhou, Multifocality as a marker of aggressiveness in medullary thyroid carcinoma: a retrospective cohort analysis of lymph node metastasis and recurrence, *The Oncologist*, Volume 31, Issue 4, April 2026, https://doi.org/10.1093/oncolo/oyag065.

In July 2026, a reader notified the Journal of concerns regarding the accuracy of the data presented in the article. The journal has contacted the authors and is investigating these concerns in line with COPE guidance. The journal is publishing this Expression of Concern to alert readers while the outcome of the investigation is pending. In the interim, the Editors advise readers to examine the details of this study with particular care.

