# Supplementary material for: Multifocality as a marker of aggressiveness in medullary thyroid carcinoma: a retrospective cohort analysis of lymph node metastasis and recurrence
Source: Oncologist. 2026 Feb 28;31(4):oyag065. doi: 10.1093/oncolo/oyag065 (PMC12990299; doi:10.1093/oncolo/oyag065)
Supplement: oyag065_Supplementary_Data [file oyag065_supplementary_data.docx]

Supplementary materials

Table S1 VIF and inter-variable correlations in the LNM regression model​​

| Variable | Unstandardized Coefficient (B) | Standard Error | Standardized Coefficient (Beta) | *t* | *p* | Zero-order correlation | Partial correlation | Tolerance | VIF |
| --- | --- | --- | --- | --- | --- | --- | --- | --- | --- |
| constant | 0.149 | 0.105 |  | 1.412 | 0.16 |  |  |  |  |
| Pre-operative calcitonin (ng/L) | 0 | 0 | 0.175 | 2.169 | 0.031 | 0.409 | 0.16 | 0.586 | 1.706 |
| Multifocality | 0.135 | 0.073 | 0.124 | 1.847 | 0.066 | 0.296 | 0.137 | 0.849 | 1.178 |
| Capsular Invasion | 0.335 | 0.073 | 0.344 | 4.591 | 0 | 0.496 | 0.325 | 0.683 | 1.464 |
| Intrathyroidal Dissemination | 0.066 | 0.107 | 0.041 | 0.615 | 0.539 | 0.225 | 0.046 | 0.86 | 1.162 |
| Lymphovascular Invasion | 0.065 | 0.099 | 0.045 | 0.657 | 0.512 | 0.266 | 0.049 | 0.809 | 1.237 |
| T Stage | 0.026 | 0.049 | 0.042 | 0.532 | 0.596 | 0.356 | 0.04 | 0.602 | 1.661 |

Table S2 VIF and inter-variable correlations in the ​​LNM (TTD + bilaterality) submodel​​

| Variable | Unstandardized Coefficient (B) | Standard Error | Standardized Coefficient (Beta) | *t* | *p* | Zero-order correlation | Partial correlation | Tolerance | VIF |
| --- | --- | --- | --- | --- | --- | --- | --- | --- | --- |
| constant | 0.282 | 0.071 |  | 3.995 | 0 |  |  |  |  |
| Pre-operative calcitonin (ng/L) | 0 | 0 | 0.215 | 2.469 | 0.014 | 0.409 | 0.182 | 0.511 | 1.956 |
| Capsular Invasion | 0.339 | 0.074 | 0.348 | 4.607 | 0 | 0.496 | 0.326 | 0.681 | 1.468 |
| Intrathyroidal Dissemination | 0.068 | 0.111 | 0.043 | 0.614 | 0.54 | 0.225 | 0.046 | 0.805 | 1.243 |
| Lymphovascular Invasion | 0.08 | 0.101 | 0.055 | 0.789 | 0.431 | 0.266 | 0.059 | 0.795 | 1.259 |
| T Stage | 0.056 | 0.059 | 0.09 | 0.937 | 0.35 | 0.356 | 0.07 | 0.424 | 2.361 |
| TTD(mm) | -0.002 | 0.003 | -0.092 | -0.871 | 0.385 | 0.295 | -0.065 | 0.349 | 2.865 |
| Bilaterality | 0.128 | 0.095 | 0.102 | 1.34 | 0.182 | 0.195 | 0.1 | 0.672 | 1.488 |

Table S3 VIF and inter-variable correlations in the ​​LNM (tumor foci + maximum diameter​​) submodel​​

| Variable | Unstandardized Coefficient (B) | Standard Error | Standardized Coefficient (Beta) | *t* | *p* | Zero-order correlation | Partial correlation | Tolerance | VIF |
| --- | --- | --- | --- | --- | --- | --- | --- | --- | --- |
| constant | 0.237 | 0.09 |  | 2.649 | 0.009 |  |  |  |  |
| Pre-operative calcitonin (ng/L) | 0 | 0 | 0.213 | 2.407 | 0.017 | 0.409 | 0.178 | 0.495 | 2.022 |
| Capsular Invasion | 0.335 | 0.074 | 0.344 | 4.528 | 0 | 0.496 | 0.321 | 0.672 | 1.487 |
| Intrathyroidal Dissemination | 0.078 | 0.109 | 0.048 | 0.714 | 0.476 | 0.225 | 0.053 | 0.843 | 1.187 |
| Lymphovascular Invasion | 0.06 | 0.101 | 0.042 | 0.597 | 0.551 | 0.266 | 0.045 | 0.798 | 1.254 |
| T Stage | 0.046 | 0.059 | 0.074 | 0.776 | 0.439 | 0.356 | 0.058 | 0.43 | 2.328 |
| Maximum Diameter (mm) | -0.002 | 0.003 | -0.059 | -0.635 | 0.526 | 0.236 | -0.048 | 0.443 | 2.256 |
| Tumor Foci | 0.053 | 0.05 | 0.072 | 1.063 | 0.289 | 0.231 | 0.079 | 0.856 | 1.168 |

Table S4 VIF and inter-variable correlations in the ​​recurrence regression model​​

| Variable | Unstandardized Coefficient (B) | | Standard Error | Standardized Coefficient (Beta) | *t* | *p* | Zero-order correlation | Partial correlation | Tolerance | VIF |
| --- | --- | --- | --- | --- | --- | --- | --- | --- | --- | --- |
| constant | | -0.152 | 0.085 |  | -1.785 | 0.076 |  |  |  |  |
| Pre-operative calcitonin (ng/L) | | 2.2E-05 | 0 | 0.045 | 0.497 | 0.62 | 0.311 | 0.038 | 0.511 | 1.956 |
| Capsular Invasion | | 0.024 | 0.063 | 0.032 | 0.377 | 0.707 | 0.283 | 0.028 | 0.572 | 1.747 |
| Intrathyroidal Dissemination | | 0.215 | 0.085 | 0.177 | 2.522 | 0.013 | 0.335 | 0.187 | 0.84 | 1.19 |
| Lymphovascular Invasion | | 0.067 | 0.082 | 0.061 | 0.813 | 0.417 | 0.267 | 0.061 | 0.742 | 1.348 |
| Calcitonin (ng/L), POD7 | | -7E-07 | 0 | -0.001 | -0.006 | 0.995 | 0.354 | 0 | 0.258 | 3.88 |
| Calcitonin (ng/L), POM3 | | 0 | 0 | 0.213 | 1.707 | 0.09 | 0.385 | 0.128 | 0.265 | 3.771 |
| T Stage | | 2.9E-06 | 0.033 | 0 | 0 | 1 | 0.277 | 0 | 0.552 | 1.81 |
| N Stage | | 0.052 | 0.035 | 0.127 | 1.496 | 0.137 | 0.338 | 0.112 | 0.573 | 1.745 |
| Multifocality | | 0.128 | 0.059 | 0.154 | 2.174 | 0.031 | 0.328 | 0.162 | 0.822 | 1.216 |
| Maximum Diameter ＞1cm | | 0.009 | 0.062 | 0.011 | 0.141 | 0.888 | 0.143 | 0.011 | 0.725 | 1.379 |

*Abbreviations:* POD7, Postoperative Day 7; POM3, Postoperative Month 3.

Table S5 VIF and inter-variable correlations in the ​​recurrence (TTD + bilaterality) submodel​​

| Variable | Unstandardized Coefficient (B) | Standard Error | Standardized Coefficient (Beta) | *t* | *p* | Zero-order correlation | Partial correlation | Tolerance | VIF |
| --- | --- | --- | --- | --- | --- | --- | --- | --- | --- |
| constant | -0.02 | 0.051 |  | -0.391 | 0.696 |  |  |  |  |
| Pre-operative calcitonin (ng/L) | 8.9E-06 | 0 | 0.018 | 0.196 | 0.845 | 0.311 | 0.015 | 0.486 | 2.057 |
| Capsular Invasion | 0.036 | 0.063 | 0.048 | 0.562 | 0.575 | 0.283 | 0.042 | 0.572 | 1.749 |
| Intrathyroidal Dissemination | 0.215 | 0.088 | 0.177 | 2.434 | 0.016 | 0.335 | 0.181 | 0.794 | 1.26 |
| Lymphovascular Invasion | 0.078 | 0.082 | 0.071 | 0.95 | 0.343 | 0.267 | 0.072 | 0.745 | 1.342 |
| Calcitonin (ng/L), POD7 | 1.5E-07 | 0 | 0 | 0.001 | 0.999 | 0.354 | 0 | 0.253 | 3.954 |
| Calcitonin (ng/L), POM3 | 0 | 0 | 0.221 | 1.733 | 0.085 | 0.385 | 0.13 | 0.257 | 3.887 |
| T Stage | -0.02 | 0.039 | -0.054 | -0.527 | 0.599 | 0.277 | -0.04 | 0.403 | 2.481 |
| N Stage | 0.057 | 0.035 | 0.141 | 1.651 | 0.101 | 0.338 | 0.124 | 0.572 | 1.747 |
| TTD(mm) | 0.003 | 0.002 | 0.126 | 1.131 | 0.26 | 0.325 | 0.085 | 0.339 | 2.948 |
| Bilaterality | 0.042 | 0.077 | 0.044 | 0.546 | 0.586 | 0.246 | 0.041 | 0.639 | 1.564 |

*Abbreviations:* POD7, Postoperative Day 7; POM3, Postoperative Month 3.

Table S6 VIF and inter-variable correlations in the ​​recurrence (tumor foci + maximum diameter​​) submodel​​

| Variable | Unstandardized Coefficient (B) | Standard Error | Standardized Coefficient (Beta) | *t* | *p* | Zero-order correlation | Partial correlation | Tolerance | VIF |
| --- | --- | --- | --- | --- | --- | --- | --- | --- | --- |
| constant | -0.119 | 0.069 |  | -1.717 | 0.088 |  |  |  |  |
| Pre-operative calcitonin (ng/L) | 8.5E-06 | 0 | 0.017 | 0.184 | 0.854 | 0.311 | 0.014 | 0.467 | 2.141 |
| Capsular Invasion | 0.029 | 0.063 | 0.039 | 0.46 | 0.646 | 0.283 | 0.035 | 0.569 | 1.756 |
| Intrathyroidal Dissemination | 0.207 | 0.086 | 0.17 | 2.407 | 0.017 | 0.335 | 0.179 | 0.827 | 1.209 |
| Lymphovascular Invasion | 0.079 | 0.081 | 0.072 | 0.966 | 0.335 | 0.267 | 0.073 | 0.749 | 1.335 |
| Calcitonin (ng/L), POD7 | 2.5E-05 | 0 | 0.026 | 0.204 | 0.838 | 0.354 | 0.015 | 0.26 | 3.851 |
| Calcitonin (ng/L), POM3 | 0 | 0 | 0.19 | 1.516 | 0.131 | 0.385 | 0.114 | 0.264 | 3.791 |
| T Stage | -0.019 | 0.037 | -0.049 | -0.504 | 0.615 | 0.277 | -0.038 | 0.44 | 2.275 |
| N Stage | 0.056 | 0.034 | 0.138 | 1.633 | 0.104 | 0.338 | 0.123 | 0.575 | 1.739 |
| Maximum Diameter (mm) | 0.003 | 0.003 | 0.094 | 1.009 | 0.314 | 0.228 | 0.076 | 0.48 | 2.085 |
| Tumor Foci | 0.087 | 0.04 | 0.154 | 2.175 | 0.031 | 0.314 | 0.162 | 0.823 | 1.215 |

*Abbreviations:* POD7, Postoperative Day 7; POM3, Postoperative Month 3.
